# Over-Expression of a Maize *N*-Acetylglutamate Kinase Gene (*ZmNAGK*) Improves Drought Tolerance in Tobacco

**DOI:** 10.3389/fpls.2018.01902

**Published:** 2019-01-04

**Authors:** Weijuan Liu, Yang Xiang, Xiaoyun Zhang, Gaoqiang Han, Xiujuan Sun, Yu Sheng, Jingwei Yan, Henrik Vibe Scheller, Aying Zhang

**Affiliations:** ^1^College of Life Sciences, Nanjing Agricultural University, Nanjing, China; ^2^Environmental Genomics and Systems Biology Division, Joint Bioenergy Institute, Lawrence Berkeley National Laboratory, Berkeley, CA, United States; ^3^State Key Laboratory of Crop Genetics and Germplasm Enhancement, Nanjing Agricultural University, Nanjing, China

**Keywords:** drought stress, *ZmNAGK*, tobacco, abiotic stress, arginine, NO

## Abstract

Water deficit is a key limiting factor that affects the growth, development and productivity of crops. It is vital to understand the mechanisms by which plants respond to drought stress. Here an *N*-acetylglutamate kinase gene, *ZmNAGK*, was cloned from maize (*Zea mays*). *ZmNAGK* was expressed at high levels in maize leaves and at lower levels in root, stem, female flower and male flower. The expression of *ZmNAGK* was significantly induced by PEG, NaCl, ABA, brassinosteroid and H_2_O_2_. The ectopic expression of *ZmNAGK* in tobacco resulted in higher tolerance to drought compared to plants transformed with empty vector. Further physiological analysis revealed that overexpression of *ZmNAGK* could enhance the activities of antioxidant defense enzymes, and decrease malondialdehyde content and leakage of electrolyte in tobacco under drought stress. Moreover, the *ZmNAGK* transgenic tobacco accumulated more arginine and nitric oxide (NO) than control plants under drought stress. In addition, the *ZmNAGK* transgenic tobaccos activated drought responses faster than vector-transformed plants. These results indicate that *ZmNAGK* can play a vital role in enhancing drought tolerance by likely affecting the arginine and NO accumulation, and *ZmNAGK* could be involved in different strategies in response to drought stress.

## Introduction

Along with global growing population and climate change, water resource scarcity is one of the great environmental challenges of our time. Plants encounter many abiotic stresses that affect growth and development, and water deficit is one of the main factors that ultimately lead to substantial yield reduction (Boyer, 1982). Thus, it is crucial to understand the mechanisms of plant response to drought (Daryanto et al., 2016). A broad range of strategies at physiological and molecular levels have been developed in plants to help them adapt to stress (Mazzucotelli et al., 2008). Abscisic acid (ABA) plays a vital role in modulating the expression of stress-related genes and cellular responses to drought stress (Todaka et al., 2017). Exogenous 24-epiBL (24-epibrassionlide) can improve the drought tolerance by reducing lipid peroxidation, plasma membrane penetration and enhancing antioxidant protective enzyme activity in *Chorispora bungeana* (Li et al., 2012). Application of exogenous BL can alleviate the detrimental effects caused by drought stress by enhancing enzymatic antioxidant enzyme activities in maize (Anjum et al., 2011). Transcription factors, like NAC (Zhu et al., 2016), DREB (Kudo et al., 2017), and WRKY (Chen et al., 2017) and signal factors, like hydrogen peroxide (H_2_O_2_), NO and Ca^2+^, are involved in drought stress tolerance in plants.

Nitric oxide (NO), as one of the second messengers, plays important roles in various physiological processes in plants, including development, defense responses, hormone responses and abiotic stress responses (Neill et al., 2003; Besson-Bard et al., 2008; Su et al., 2018). NO could increase the activities of antioxidant defense system enzymes, such as ascorbate peroxidase (APX), catalase (CAT), glutathione reductase (GR) and superoxide dismutase (SOD), to enhance the drought tolerance as a second messenger (Zhang et al., 2007; Shan et al., 2015; Peng et al., 2016; Hasanuzzaman et al., 2018). It is therefore important to understand the mechanisms of NO production and function. The biosynthesis of NO in plants has been difficult to resolve and there appears to be several different pathways. One of the enzymatic biosynthesis pathways of NO is the L-Arginine (Arg) dependent pathway using an NO synthase like enzyme (Besson-Bard et al., 2008; Su et al., 2018; Zhao et al., 2018). Arginine is not only an essential amino acid in the process of protein synthesis but also an intermediate for nitrogen storage (Micallef and Shelp, 1989; Llacer et al., 2008), and a precursor of polyamines (Takahashi and Kakehi, 2010) and NO (Crawford, 2006).

The first step of the Arg biosynthesis pathway is the conversion of acetyl-CoA and glutamate to *N*-acetylglutamate (NAG) catalyzed by *N*-acetylglutamate synthase (NAGS). Overexpression of a tomato *NAGS* gene (*SlNAGS1*) in *Arabidopsis thaliana* resulted in high ornithine levels and increased tolerance to salt and drought stress (Kalamaki et al., 2009). The second step of Arg biosynthesis is catalyzed by *N*-acetylglutamate kinase (NAGK). NAGK is the target of Arg in the negative feedback loop of the Arg biosynthetic pathway (McKay and Shargool, 1981). NAGK in plants has been identified in several studies (Burillo et al., 2004; Slocum, 2005; Chen et al., 2006; Winter et al., 2015), and many roles of NAGK in plants have been reported. NAGK was found to modulate the balance of nitrogen and carbon by interacting with PII signaling proteins (Winter et al., 2015), and OsNAGK1 could interact with PII-like protein (OsGlnB) in rice (Sugiyama et al., 2004). AtNAGK is implicated in gametophyte function and embryo development in *A. thaliana* (Huang et al., 2017). Since NAGK is an indirect enzyme leading to NO biosynthesis pathway via arginine, we hypothesized that NAGK similarly to what has been shown for NAGS could have a function in response to drought stress.

In this study, we identified a NAGK from maize, ZmNAGK, by sequence alignment and phylogenetic analysis. Overexpression of *ZmNAGK* in tobacco resulted in increased tolerance to drought stress. Moreover, *ZmNAGK* overexpressing plants accumulated more arginine and NO in response to drought than the control plants.

## Materials and Methods

### Plant Materials and Treatments

Seeds of maize (*Z. mays* cv Nongda 108, from Nanjing Agricultural University, China) were grown in greenhouse at a temperature of 22 to 28°C, photosynthetic active radiation of 200 μmolm^-2^ s^-1^, a photoperiod of 14/10 h (day/night) and watered every day.

Maize plants were excised at the base of stem and placed in distilled water for 2 h to eliminate the effect from wound stress. Then the detached plants were placed in beakers containing 10% (w/v) polyethyleneglycol (PEG6000) solution, 100 mM NaCl, 100 μM ABA, 50 nM BR, 10 mM H_2_O_2_ or distilled water (as control), respectively. Beakers were wrapped with aluminum foil. Detached plants treated with distilled water under the same conditions at the same time served as controls for the above. The second leaves from three maize seedlings were sampled at the time points indicated and immediately frozen under liquid N_2_ for further analysis. The experiments were repeated at least three times.

Seeds of maize were sowed in the soil. When the second leaf was fully expanded, the root, second leaf (as the young leaf) and stem were collected and frozen under liquid N_2_. The eighth leaf from the stem base (as the old leaf) was collected and the male or female flowers were collected at the pollen stage. Total RNA was extracted from these tissues and used to detect the *ZmNAGK* expression.

The seeds of *Nicotiana tabacum* were sown into pots that were filled with an equal quantity of moisture and soil in green house maintained at 25°C under a 16/8 h (day/night) photoperiod. After 4 weeks, tobacco seedlings were used to do phenotypic analysis. Leaves (L1–L4 from the top) from the transgenic and vector-transformed plants were sampled to analyze the relative water content (RWC) (Hu et al., 2013), malondialdehyde (MDA), electrolyte leakage, activities of antioxidant enzymes, arginine content and NO content. Leaves from three tobacco plants were used to measure these physiological parameters and the experiments were repeated at least three times.

### Isolation of the Maize *ZmNAGK* Gene

A blast search with rice *OsNAGK1* sequence as a query from NCBI database revealed good homology with the product of the predicted maize gene. *ZmNAGK* is also known as GRMZM2G132777 in MaizeGDB. Specific primers (*ZmNAGK*-F and *ZmNAGK*-R, see Supplementary Table S1) were designed and used to amplify the coding sequence by PCR. The PCR product was purified and subcloned into the pMD19-T vector (Takara, Dalian, China) for sequence verification.

### Alignment and Phylogenetic Analysis

Sequences of plant NAGK proteins from different species were retrieved from NCBI and aligned by CLUSTAL W^1^. Identical and similar residues were shaded by BOXSHADE^2^. The putative plastid-target sequence was predicted by the iPSORT^3^ (Sugiyama et al., 2004). A phylogeny tree was constructed based on the sequence alignment using the Neighbor-Joining method and bootstrap analysis was performed with 1000 using the MEGA 6.0 software.

### Isolation of Total RNA and qRT-PCR Analysis

Total RNA was isolated from maize or tobacco leaves using an RNAiso Plus kit (TaKaRa) following the manufacturer’s protocol and the cDNA was synthesized by the 5xAll-In-One MasterMix with AccuRT Genomic DNA Removal Kit (abm, Zhenjiang, China). Transcript levels of *ZmNAGK* were measured by qRT-PCR using a DNA Engine Opticon 2 realtime PCR detection system (Bio-Rad) with EvaGreen 2X qPCR MasterMix-No Dye (abm) according to the manufacturer’s instructions. The expression level was normalized against that of *ZmActin2* in maize or *NtActin* in tobacco (*N. tabacum*). The specific primers for qRT-PCR were designed according to the relevant sequences and are shown in Supplementary Table S1.

### Generation of Transgenic Tobacco

The full-length coding sequence of *ZmNAGK* was inserted into the *KpnI*-*BamHI* sites of the binary vector 1300-221-3^∗^Flag driven by the cauliflower mosaic virus 35S promoter. Primers for constructing recombinant vector (*ZmNAGK*-P1 and *ZmNAGK*-P2) are shown in Supplementary Table S1. The recombinant vector or empty 1300-221-3^∗^Flag vector was introduced into tobacco using *Agrobacterium tumefaciens* strain GV3101 via leaf disc transformation (Horsch et al., 1985). Independent transgenic lines were obtained by hygromycin-resistance selection and confirmed by PCR in T_0_ plants. In addition, tobacco plants transformed with empty vector alone were also subjected to similar analysis. T_0_, T_1_, and T_2_ plants were grown in agreenhouse, and the presence of the transgene was confirmed in each generation by PCR analysis. The *ZmNAGK* expression in *ZmNAGK* overexpressors was detected by semi-quantitative RT-PCR analysis. The *NtUbi* (GenBank accession number: U66264) was used as an internal reference. Three independent T_2_ lines, *ZmNAGK-2, ZmNAGK-3*, and *ZmNAGK-15* were selected for further analysis.

### Water Loss Measurement and Stomatal Density

Detached leaves (L1–L4) from 4-week-old tobacco seedlings were placed at room temperature. Their fresh weight was recorded immediately and then every 15 min for 2 h. The percentage water loss was calculated as (initial fresh weight - final fresh weight)/initial fresh weight × 100% (Yoo et al., 2011).

The impression approach which was expressed as the number of stomata per unit leaf area was used to analyze leaf stomatal density (Radoglou and Jarvis, 1990). The abaxial lower epidermis of the leaf was cleaned by a degreased cotton ball, and then carefully scraped with scalpel. The thin film (approximately 5 mm^∗^15 mm) was peeled off from the leaf surface, lied on a glass slide, immediately covered with a cover slip, and then lightly pressured with fine point tweezers. Alcohol lamp was used to gently heat the back of the glass slide to stabilize the epidermis. Numbers of stomatas for each film strip were counted under a photomicroscope system with a computer attachment (OLYMPUS BX53). Impressions were taken from the L3 and L4, fully expanded leaves for each treatment.

### Drought Tolerance and Survival Rate

Four-week-old tobacco seedlings grown in pots with soil were treated by withholding water for 10 days. The phenotype of seedlings was photographed and the relative water content was determined as described by Jiang and Zhang (2002). Subsequently, the plants were re-watered as normal for 1 week and the survival rate was calculated.

### Measurement of Malondialdehyde Content and Electrolyte Leakage

Malondialdehyde content was measured as described previously (Shi et al., 2012). About 0.1 g of tobacco leaves was used and absorbance values at 450, 532, and 600 nm were determined with a spectrophotometer. The concentration of MDA was calculated using the following formula: C (μmol/L) = 6.45^∗^ (OD_532_ - OD_600_) - 0.56^∗^ OD_450_. The percentage of electrolyte leakage was determined according to Shi et al. (2012).

### Antioxidant Enzyme Assay

Tobacco leaf samples (200 mg) were homogenized in 0.6 mL of 50 mM potassium phosphate buffer (pH 7.0) containing 1 mM EDTA and 1% polyvinylpyrrolidone, with the addition of 1 mM sodium ascorbate in the case of APX assay. The homogenate was centrifuged at 12,000 *g* for 30 min at 4°C, and the supernatant was immediately used for the subsequent antioxidant enzyme assays. The total activity of APX was measured by monitoring the decrease of oxidized ascorbate in absorbance at 290 nm as previously described (Nakano and Asada, 1981; Zhu et al., 2013). Total SOD activity was assayed by monitoring the inhibition of photochemical reduction of nitro blue tetrazolium (NBT). One unit of SOD activity was defined as the amount of enzyme required to cause 50% inhibition of the reduction of NBT as monitored at 560 nm (Giannopolitis and Ries, 1977; Jiang and Zhang, 2002).

### Measurement of Arginine

Tobacco leaves (100 mg) were extracted, derivatized, and analyzed as described by Rinder et al. (2008).

### Measurement of Nitric Oxide

The tobacco leaves of transgenic plants were homogenized in Cell and Tissue Lysis Buffer for Nitric Oxide Assay (Beyotime Institute of Biotechnology, China). The homogenate was centrifuged at 12,000 *g* for 10 min at 4°C. Total NO concentration was determined by measuring the concentration of nitrate and nitrite, a stable metabolite of NO, according to the Griess assay with Total Nitric Oxide Assay Kit (Beyotime).

## Results

### Identification and Sequence Analysis of ZmNAGK

**FIGURE 1:**
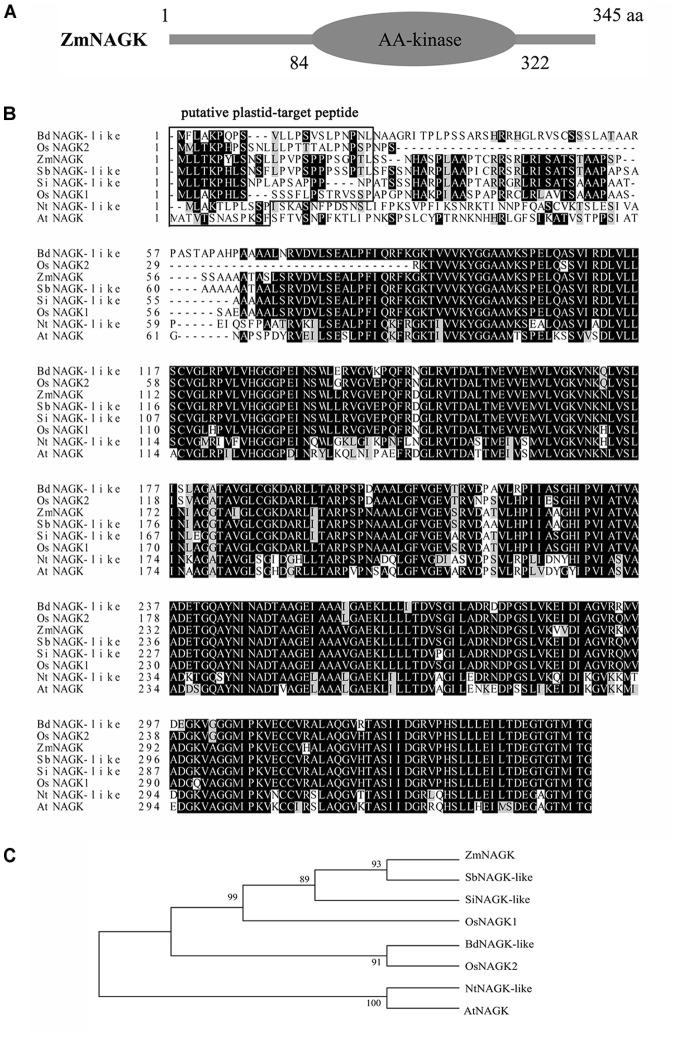
Alignment and phylogenetic analysis of ZmNAGK with NAGKs from different species. **(A)** *ZmNAGK* encodes a protein of 345 amino acid residues and contains an AA-kinase domain. **(B)** The amino-acid sequences of NAGKs from *Arabidopsis thaliana, Brachypodium distachyon, Nicotiana tabacum, Oryza sativa, Setaria italica, Sorghum bicolor*, and *Zea mays* were aligned. Identical and similar residues were shaded in black and gray, respectively. The putative plastid-target sequence, which was predicted by the iPSORT (http://ipsort.hgc.jp/) programs are boxed. **(C)** Phylogeny tree of NAGKs was calculated in the MEGA 6.0 program. The phylogenetic relationship between the examined sequences was performed using the Neighbor-Joining method with p-distance correction. Bootstrap values were derived from 1000 replicate runs. The accession numbers: AtNAGK (NP_191315.1), BdNAGK-like (XP_003570025.1), NtNAGK-like (XP_016482712.1), OsNAGK1 (XP_015633506.1), OsNAGK2 (BAD88532.1), SiNAGK-like (XP_004976436.1), SbNAGK-like (XP_002446901.2), ZmNAGK (XP_008668008.1).

The 1230 bp full-length cDNA sequence was obtained from NCBI (XP_008668008.1). It contains a 1038 bp open reading frame, which encodes a polypeptide of 345 amino acid residues. ZmNAGK contains an amino acid kinase (AA-kinase) domain (Figure 1A). Multiple sequence alignment showed that NAGK was highly conserved among *A. thaliana, Brachypodium distachyon, N. tabacum, Oryza sativa, Setaria italica, Sorghum bicolor*, and *Z. mays*, except for 60 amino acid residues at the N-terminus. The ZmNAGK protein contains a putative plastid-targeting polypeptide like the homolog proteins in rice which was reported before (Sugiyama et al., 2004) (Figure 1B). Phylogeny analysis revealed that ZmNAGK was homologous to NAGKs in *S. bicolor* and *S. italica* and to OsNAGK1 from *O. sativa* (Figure 1C).

### The Expression of *ZmNAGK* in Maize

**FIGURE 2:**
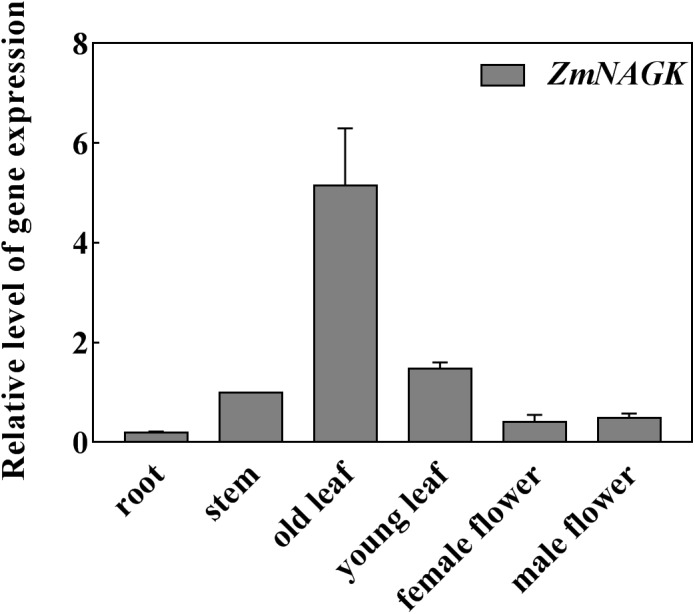
Expression of *ZmNAGK* in maize tissues. RNA was isolated from root, stem, old leaf, young leaf, female flower and male flower, and expression of *ZmNAGK* relative to *ZmActin2* determined by qRT-PCR analysis. Data are means ± SD (*n* = 3).

We assessed the expression pattern of *ZmNAGK* in various maize tissues. Total RNA was isolated from various tissues of maize including root, stem, leaf, female flower and male flower. Quantitative real-time polymerase chain reaction (qRT-PCR) analysis revealed that *ZmNAGK* was expressed in all the above tissues, and particularly in leaves (Figure 2).

**FIGURE 3:**
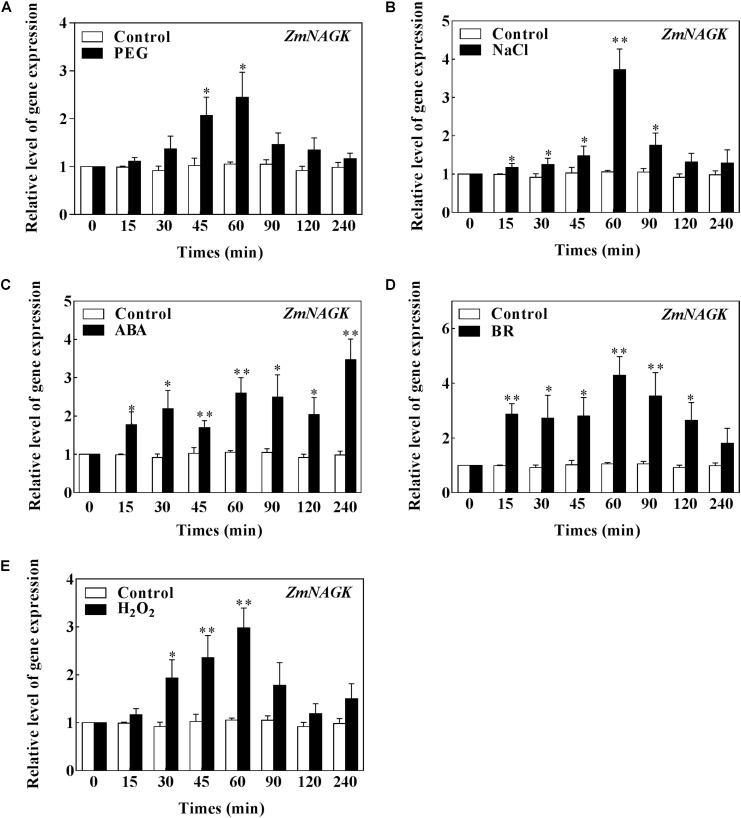
Expression of *ZmNAGK* in leaves of maize plants exposed to different treatments. The detached maize plants were treated with **(A)** 10% PEG 6000 **(B)** 100 mM NaCl **(C)** 100 μM ABA **(D)** 50 nM BR **(E)** 10 mM H_2_O_2_ for various times as indicated. Expression level of *ZmNAGK* relative to *ZmActin2* was analyzed by qRT-PCR. Data are means ± SD (*n* = 3). The asterisks indicate a significant difference compared to Control using the unpaired Student’s *t*-test (^∗^*P* < 0.05, ^∗∗^*P* < 0.01).

To understand the functions of ZmNAGK, we investigated the expression of *ZmNAGK* in maize plants under abiotic stimuli. The maize plants were treated with polyethylene glycol (PEG), NaCl, ABA, brassinosteroid (BR) or H_2_O_2_, and then the expression of *ZmNAGK* in leaves was determined by qRT-PCR assay. *ZmNAGK* expression was prominently increased after 45 min PEG treatment, 60 min after NaCl treatment with a three-fold change. ABA significant induced the expression of *ZmNAGK* at 15 min and peaked at 240 min after ABA treatment. *ZmNAGK* expression was increased at 15 min and maximized at 60 min after BR treatment in a smooth trend. H_2_O_2_ also increased the transcription of *ZmNAGK* (Figure 3). These results suggested that *ZmNAGK* may play a positive role in response to abiotic stress.

### Overexpression of *ZmNAGK* in Tobacco Enhanced Seedling Drought Stress Tolerance

To further investigate the function of *ZmNAGK* in abiotic stress tolerance, transgenic tobacco plants overexpressing *ZmNAGK* under control of the cauliflower mosaic virus 35S promoter were generated. The recombinant vector and empty vector were transferred to *Agrobacterium tumefaciens* and then were used to transform tobacco. Three independent lines (*ZmNAGK-2, ZmNAGK-3, ZmNAGK-15*) with *ZmNAGK* expression (Supplementary Figure S1) analyzed by semi-qRT-PCR were selected for further analysis. No visible phenotypic alteration was observed between the three transgenic and the vector-transformed lines under normal growth conditions (Supplementary Figure S2).

**FIGURE 4:**
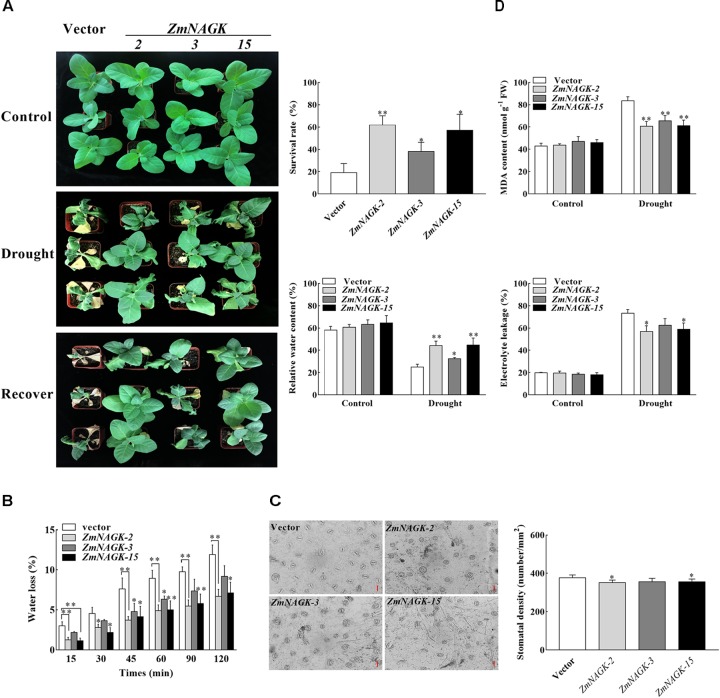
Tolerance of *ZmNAGK* transgenic tobacco to drought stress. **(A)** Phenotype of vector- or *ZmNAGK*-transformed plants grown on soil and treated by withholding water for 10 days and re-watered for 1 week. Control plants were watered daily throughout the experiment. The survival rate of tobacco plants after re-watering for 1 week. The relative water content in the leaves of vector- or *ZmNAGK*-transformed plants under drought treatment for 10 days. **(B)** Water loss of tobacco leaves at room temperature. Detached tobacco leaves from transgenic and vector-transformed seedlings were placed at room temperature for 2 h and the percentage of water loss was recorded at different time points. **(C)** Stomatal density on leaf epidermis of overexpressors and vector-transformed seedlings. **(D)** Determination of the MDA content and electrolyte leakage in transgenic and vector-transformed plants under water deficit. The content of MDA and the electrolyte leakage were determined in the leaves of *ZmNAGK*- and vector-transformed plants under drought treatment for 7 days and in well-watered control plants. Data are means ± SD (*n* = 3). The asterisks indicate a significant difference compared to vector-transformed plants using the unpaired Student’s *t*-test (^∗^*P* < 0.05, ^∗∗^*P* < 0.01).

Phenotypic analysis showed that the *ZmNAGK* transgenic lines displayed an improvement in drought tolerance compared to vector-transformed plants (Figure 4A). The seedlings of vector-transformed tobacco became severely wilted and impaired after 10 days drought stress, while *ZmNAGK* transgenic plants showed more open, green leaves (Figure 4A). Following the dehydration treatment, the plants were re-watered for a week to determine their survival rate, and the *ZmNAGK* transgenic plants had a significantly higher survival rate than the vector-transformed plants (Figure 4A). Moreover, under normal conditions, there was no significant difference in relative water content (RWC) between *ZmNAGK* transgenic lines and vector-transformed plants, but under drought conditions, *ZmNAGK* transgenic plants showed higher RWC compared with vector-transformed plants (Figure 4A). In view of the above results, the ability of water retention in transgenic tobacco was further investigated. Water loss rate and stomatal density from detached leaves was measured. We found that the detached leaves of the *ZmNAGK* transgenic tobaccos lost water at a lower rate (Figure 4B) and had minimal lower stomatal density (Figure 4C) than those of the vector-transformed plants. This result suggests *ZmNAGK* improves the water retention ability of tobacco. These data indicated that overexpression of *ZmNAGK* in tobacco enhanced tolerance to drought stress.

### Overexpression of *ZmNAGK* in Tobacco Alleviated Oxidative Damage to Seedlings Under Drought Stress

Tolerance of plants under different stresses has been shown to be correlated with change in MDA content (Sharma et al., 2011; Cheng et al., 2013). MDA content increased when plants were subjected to drought stress (Lim and Lee, 2014). To further investigate the effect of *ZmNAGK* on oxidative damage to seedlings under drought stress, MDA content and electrolyte leakage were studied. In the absence of stress, there was no difference between the plants in MDA content, and in response to drought the MDA content increased in all the lines (Figure 4D). However, the MDA content in three transgenic lines, *ZmNAGK-2, ZmNAGK-3*, and *ZmNAGK-15*, accumulated less in response to drought compared to the vector-transformed plants (Figure 4D). Similarly, when plants were exposed to drought stress, the electrolyte leakage was slightly lower in *ZmNAGK* transgenic lines than in vector-transformed plants (Figure 4D). These results could suggest that overexpression of *ZmNAGK* in tobacco alleviates oxidative damage to seedlings under drought stress.

### Overexpression of *ZmNAGK* in Tobacco Enhanced the Capacity of Antioxidant Defense

**FIGURE 5:**
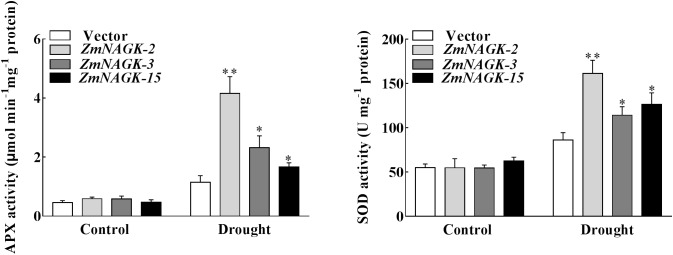
Analysis of antioxidant enzyme activities in drought-induced *ZmNAGK* transgenic and vector-transformed tobaccos. The activities of APX and SOD were measured in leaves of seedlings treated with distilled water (Control) or drought for 7 days. Data are means ± SD (*n* = 3). The asterisks indicate a significant difference compared to vector-transformed plants using the unpaired Student’s *t*-test (^∗^*P* < 0.05, ^∗∗^*P* < 0.01).

To further assess the role of *ZmNAGK* in alleviating oxidative damage, the activities of antioxidant enzymes ascorbate peroxidase (APX) and superoxide dismutase (SOD) were analyzed. Under normal condition, no significant differences in APX and SOD activities were detected between *ZmNAGK* transgenic lines and vector-transformed plants. However, when the plants were exposed to drought stress, *ZmNAGK* transgenic plants had markedly higher APX and SOD activities than vector-transformed plants (Figure 5), especially in *ZmNAGK-2*. These results could suggest that *ZmNAGK* increases the capacity of antioxidant defense in tobacco under drought stress.

### Overexpression of *ZmNAGK* in Tobacco Increased Arginine and NO Content Under Drought Stress

**FIGURE 6:**
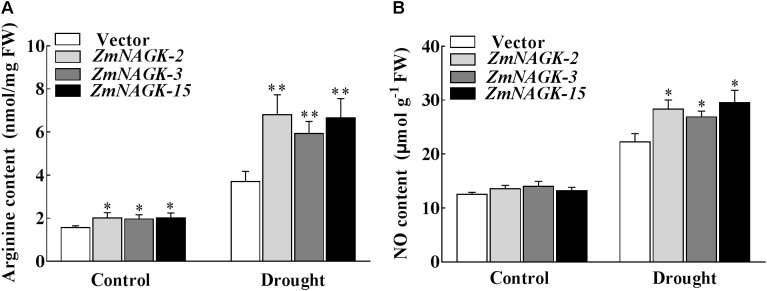
The content of arginine and NO in the leaves of *ZmNAGK* transgenic and vector-transformed plants in response to drought stress. The seedlings were treated with distilled water (Control) or drought for 7 days, and then the content of arginine **(A)** and NO **(B)** in leaves was analyzed. Data are means ± SD (*n* = 3). The asterisks indicate a significant difference compared to vector-transformed plants using the unpaired Student’s *t*-test (^∗^*P* < 0.05, ^∗∗^*P* < 0.01).

Arg is a precursor of NO (Crawford, 2006), and NAGK affects Arg biosynthesis (McKay and Shargool, 1981; Slocum, 2005; Winter et al., 2015; Huang et al., 2017). *ZmNAGK* transgenic lines accumulated more arginine compared with vector-transformed plants under drought stress (Figure 6A). Consistently, *ZmNAGK* transgenic lines accumulated slightly more NO compared with vector-transformed plants under drought stress (Figure 6B). This suggests that *ZmNAGK* can increase arginine production and NO level in tobacco under drought stress.

### Overexpression of *ZmNAGK* in Tobacco Activated the Expressions of Abiotic Stress-Response Genes Under Drought Stress

**FIGURE 7:**
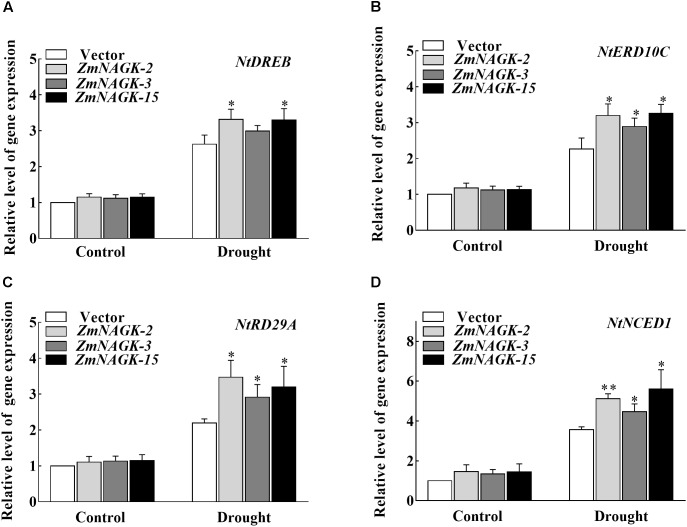
Expressions of stress-related genes in leaves of *ZmNAGK* transgenic and vector-transformed plants in response to drought stress. **(A–D)** The transcript levels of *NtDREB, NtERD10C, NtRD29A*, and *NtNCED1* under normal conditions or after 7 days drought treatment in transgenic and vector-transformed seedlings, respectively. Expression levels of these genes were relative to *NtActin* was analyzed by qRT-PCR. Data are means ± SD (*n* = 3). The asterisks indicate a significant difference compared to vector-transformed plants using the unpaired Student’s *t*-test (^∗^*P* < 0.05, ^∗∗^*P* < 0.01).

It is reported that the expression of the gene for *ERD* (early responsive to dehydration) is strongly induced by dehydration stress (Nakashima et al., 1997; Simpson et al., 2003). Overexpression of *DREB1* (dehydration-responsive element binding protein) and *NCED* (9-*cis*-epoxycarotenoid dioxygenase), respectively, in Arabidopsis transgenic plants increased stress tolerance to drought (Liu et al., 1998; Iuchi et al., 2001). To investigate the putative molecular mechanisms of *ZmNAGK* function in drought stress resistance in plants, the expressions of several stress-responsive genes in transgenic and vector-transformed plants were analyzed via qRT-PCR assay. Our results showed that the expression levels of several stress-responsive marker genes such as *NtDREB, NtERD10C, NtRD29A*, and *NtNCED1* were increased under drought stress in control plants, and more importantly in transgenic tobaccos (Figure 7).

## Discussion

As sessile organisms, plants are always subjected to various abiotic stresses which cause severe damage to growth, physiology and reproduction. Maize is one of the main crops where the yield is often threatened by drought stress. A recent study concluded that drought stress globally led to up to 40% yield reduction of maize from 1980 to 2015 (Daryanto et al., 2016). Therefore, it is essential to obtain genes related to drought tolerance, understand the mechanisms of plant drought tolerance and cultivate drought-tolerant crops (Wang et al., 2003; Vinocur and Altman, 2005; Valliyodan and Nguyen, 2006; Cattivelli et al., 2008). In the present study, we identified a *NAGK* in maize, *ZmNAGK*. ZmNAGK possesses a putative plastid-target peptide at N-terminus and an AA-kinase domain as has been reported in OsNAGKs and AtNAGK (Sugiyama et al., 2004). In the phylogenetic analysis, ZmNAGK had very high sequence similarity to NAGKs from other plants, and was clustered with OsNAGK1 and NAGKs from *S. italica* and *S. bicolor*, suggesting a comparable function for these proteins among plant species. However, little is known about the functions of NAGK in abiotic stress.

In this report, we demonstrated that *ZmNAGK* gene played a positive role in response to drought stress from the following evidence: First, the phytohormones BR and ABA can regulate the protective responses to various stresses in plants (Jiang and Zhang, 2002; Xia et al., 2009; Zhang et al., 2011). Here the increased expression of *ZmNAGK* under different treatments indicated that *ZmNAGK* expression was positively correlated with response to abiotic stimuli in maize. This result also suggested a possible regulatory role of *ZmNAGK* in maize exposed to drought stress. Secondly, the *ZmNAGK* transgenic lines displayed enhanced tolerance to drought stress, higher survival rate and higher water content under drought stress. Thirdly, it is reported that water deficit is related to stomatal density in plant (Xu and Zhou, 2008). Over-expression of *ZmNAGK* in transgenic tobacco led to higher water retention ability. Fourthly, drought stress induces ROS production and overproduced ROS results in oxidative damage in plants. Over-expression of *ZmNAGK* in transgenic tobacco could relieve the oxidative damage in response to drought stress. Fifthly, over-expression of *ZmNAGK* in transgenic tobacco improved the antioxidant defense ability of plants under drought stress. And stress-responsive genes such as *NtDREB, NtRD29A, NtERD10C* could be activated in *ZmNAGK* transgenic tobaccos under drought stress. Kalamaki et al. (2009) demonstrated that overexpression of tomato *N*-acetyl-L-glutamate synthase gene (*NAGS1*), which catalyzes the first step in arginine biosynthesis, increased tolerance to salt and drought stresses in *A. thaliana*, implying arginine biosynthesis was involved with stress tolerance of plants. We found that over-expression of *ZmNAGK* in transgenic tobacco accumulated more arginine under drought stress. Our findings in this study, combined with previous reports, suggest that ZmNAGK, which catalyzes the second step in arginine biosynthesis, would play a significant role in response to drought stress.

Nitric oxide, as a key signaling molecule, plays an essential role in the activation of plant defense signaling pathways in response to various abiotic stresses (Wang and Yang, 2005). Drought stress can induce NO generation (Gould et al., 2003; Neill et al., 2003), which enhances the water stress tolerance of plants (Garcia-Mata and Lamattina, 2001; Farooq et al., 2009). We measured the NO content before and after drought stress in tobacco, and the NO content in *ZmNAGK* transgenic tobaccos increased after drought stress compared to the vector-transformed plants. Winter et al. (2015) have reported that arginine is an important precursor of NO. Since NAGK participates in arginine biosynthesis (Shargool et al., 1988; Kalamaki et al., 2009; Huang et al., 2017), the enhanced drought stress tolerance in *ZmNAGK* expressing plants could be related to the NO accumulation via the arginine biosynthesis pathway. However, additional mechanism of the *ZmNAGK-*induced drought tolerance cannot be excluded, and more details of NO production and arginine metabolism need to be explored.

In addition, it is reported that under stress, the expression levels *NtDREB, NtRD29A* (response to dehydration), *NtERD10C* were significantly up-regulated in transgenic tobacco of a *FvMYB1* gene from *Fraxinus velutina* (Li et al., 2016). The increased gene expressions of stress-related genes in *ZmNAGK* transgenic tobaccos indicated there are different targets downstream NO to improve drought stress tolerance.

## Conclusion

We identified and characterized a NAGK in maize, ZmNAGK. Overexpression of *ZmNAGK* gene improved drought tolerance in tobacco by higher water retention, antioxidant defense ability, lower oxidative damage and accumulated more arginine. Moreover, the drought tolerance of *ZmNAGK* transgenic tobacco could be related at least in part to NO production.

## Author Contributions

AZ conceived the project and designed the experiments. WL did most of the experiments and analysis presented in this study and wrote the manuscript. YX helped with phenotypic analysis and generated Figure 1. XZ generated Figure 6 and Supplementary Figure S1. GH participated in analysis of activities of antioxidant enzymes. XS measured the water loss rate of Figure 4. YS cloned the gene. JY gave advice to manuscript writing. HS revised the manuscript. All authors approved the final manuscript.

## Conflict of Interest Statement

The authors declare that the research was conducted in the absence of any commercial or financial relationships that could be construed as a potential conflict of interest.

## Footnotes

1 http://clustalw.genome.ad.jp/

2 http://www.ch.embnet.org/software/BOX_form.html

3 http://ipsort.hgc.jp/
