# Supplementary material for: Over-Expression of a Maize N-Acetylglutamate Kinase Gene (ZmNAGK) Improves Drought Tolerance in Tobacco
Source: Front Plant Sci. 2019 Jan 4;9:1902. doi: 10.3389/fpls.2018.01902 (PMC6328498; doi:10.3389/fpls.2018.01902)
Supplement: Supplementary file 1 [file Table_1.docx]

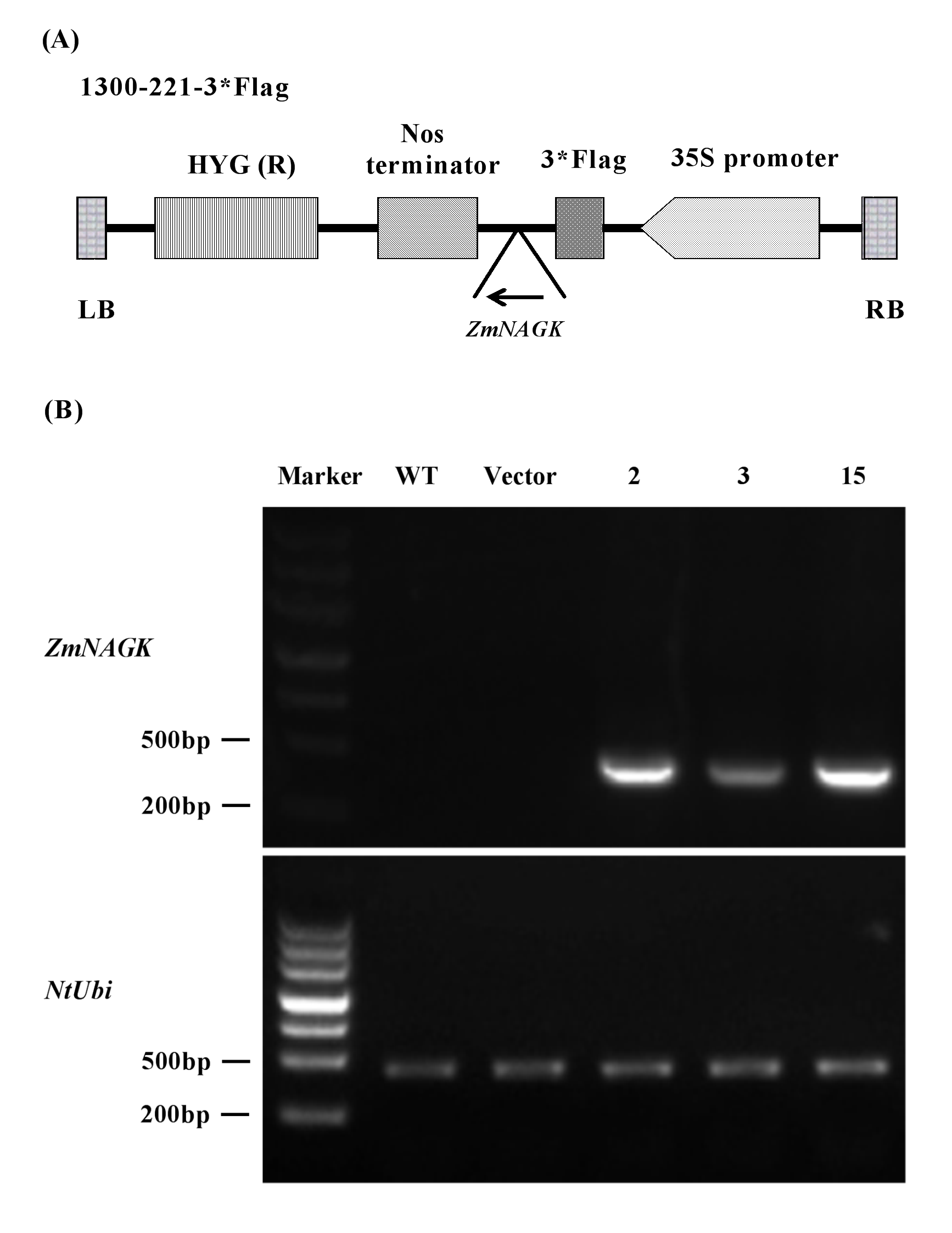


**Supplemental Figure 1** Construction of the *ZmNAGK* overexpressing transgenic plants. (A) Construction of the transgenic vector 1300-221-3*Flag-*ZmNAGK*. (B) Analysis of *ZmNAGK* expression in T_2_ vector-transformed and *ZmNAG*K (-*2*, -*3* and -*15*) tobacco plants. Expression level of *ZmNAGK* relative to *NtUbi* was analyzed by semi-qRT-PCR.


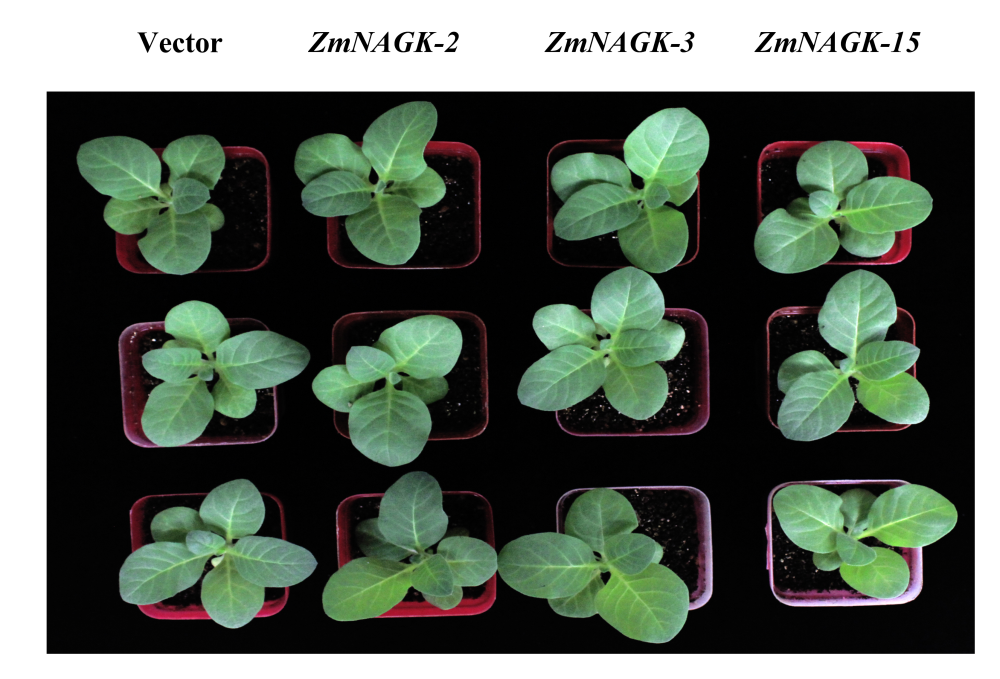


**Supplemental Figure 2** Phenotype of *ZmNAGK* transgenic and vector-transformed plants under non-stress conditions.
