# Supplementary material for: Over-Expression of a Maize N-Acetylglutamate Kinase Gene (ZmNAGK) Improves Drought Tolerance in Tobacco
Source: Front Plant Sci. 2019 Jan 4;9:1902. doi: 10.3389/fpls.2018.01902 (PMC6328498; doi:10.3389/fpls.2018.01902)
Supplement: Supplementary file 2 [file Table_2.docx]

**PCR Primers Used in the Paper**

**Genes and primers used in cloning**

| **Primer Name** | **Sequence (5’-3’)** |
| --- | --- |
| *ZmNAGK*-F | ATGGTCCTCACGAAACCCTA |
| *ZmNAGK*-R | GCCAGTGATCATGGTGCC |
| *ZmNAGK*-P1 | GGTACC ATGGTCCTCACGAAACCCTA |
| *ZmNAGK*-P2 | GGATCC GCCAGTGATCATGGTGCC |

**Genes and primers used in qRT-PCR**

| **Genes** | **Primer** | **Sequence (5’-3’)** |
| --- | --- | --- |
| *NtUbiquitin* | NtUbi-semi-F | TTAACACATGCAAGTCGGACG |
| (U66264) | NtUbi-semi-R | GAGACCTCAGTAGACAAAGCACATC |
| *NtActin* | NtActin-qF | CAAGGAAATCACCGCTTTGG |
| (U60495) | NtActin-qR | AAGGGATGCGAGGATGGA |
| *ZmActin2* | ZmActin2-qF | GCCATCCATGATCGGTATGG |
| (EU952376) | ZmActin2-qR | GTCGCACTTCATGATGGAGTTG |
|  | ZmNAGK-qF | ACTCTCAGCTCCAACCATGC |
| *ZmNAGK* | ZmNAGK-qR | ACCACCGTCTTGCCTTTGAA |
| (GRMZM2G132777) | ZmNAGK-semi-F | TTCAGCGATTCAAAGGCAAGACG |
|  | ZmNAGK-semi-R | AGACGGGCGAGCGGTGATAAGG |
| *NtDREB* | NtDREB-qF | GCCGGAATACACAGGAGAAG |
| (XM_009783485.1) | NtDREB-qR | CCAATTTGGGAACACTGAGG |
| *NtERD10C* | NtERD10C-qF | AACGTGGAGGCTACAGATCG |
| (107793843) | NtERD10C-qR | GTTCCTCTTGGGCATGAGTT |
| *NtNCED1* | NtNCED1-qF | AAGAATGGCTCCGCAAGTTA |
| (HM068892) | NtNCED2-qR | GCCTAGCAATTCCAGAGTGG |
| *NtRD29A* | NtRD29A-qF | TCGGTGTACCAACAGGCATA |
| (XM_009776867.1) | NtRD29A-qR | CCCTTGCTTTGGTGTTGTTT |
